# Sleep stage classification from heart-rate variability using long short-term memory neural networks

**DOI:** 10.1038/s41598-019-49703-y

**Published:** 2019-10-02

**Authors:** Mustafa Radha, Pedro Fonseca, Arnaud Moreau, Marco Ross, Andreas Cerny, Peter Anderer, Xi Long, Ronald M. Aarts

**Affiliations:** 1 Royal Philips, Research, High Tech Campus 34, 5656 AE Eindhoven, The Netherlands; 2 Eindhoven University of Technology, P.O. Box 513, 5600 MB Eindhoven, The Netherlands; 3 Philips Austria GmbH, Kranichberggasse 4, 1120 Vienna, Austria

**Keywords:** Sleep disorders, Biomedical engineering, Computer science

## Abstract

Automated sleep stage classification using heart rate variability (HRV) may provide an ergonomic and low-cost alternative to gold standard polysomnography, creating possibilities for unobtrusive home-based sleep monitoring. Current methods however are limited in their ability to take into account long-term sleep architectural patterns. A long short-term memory (LSTM) network is proposed as a solution to model long-term cardiac sleep architecture information and validated on a comprehensive data set (292 participants, 584 nights, 541.214 annotated 30 s sleep segments) comprising a wide range of ages and pathological profiles, annotated according to the Rechtschaffen and Kales (R&K) annotation standard. It is shown that the model outperforms state-of-the-art approaches which were often limited to non-temporal or short-term recurrent classifiers. The model achieves a Cohen’s k of 0.61 ± 0.15 and accuracy of 77.00 ± 8.90% across the entire database. Further analysis revealed that the performance for individuals aged 50 years and older may decline. These results demonstrate the merit of deep temporal modelling using a diverse data set and advance the state-of-the-art for HRV-based sleep stage classification. Further research is warranted into individuals over the age of 50 as performance tends to worsen in this sub-population.

## Introduction

Sleep is a reversible state of disconnection from the external environment characterized by reduced vigilance and quiescence. It plays an essential role in the diurnal regulation of mind and body in mammals, and is hypothesized to have a wide array of functions ranging from digestion to memory consolidation. The objective measurement of sleep in adult humans involves sleep staging: the process of segmenting a sleep period into *epochs*, typically 30 seconds long, and assigning a sleep stage to each epoch. The American Association of Sleep Medicine (AASM)^1^ distinguishes five sleep stages: rapid eye movement (REM) sleep, three levels of non-REM sleep (N1, N2, N3) and wake (W). Sleep staging is done through manual visual scoring of electro-graphic measurements of the brain, eye movement and chin muscles, measured respectively with electroencephalography (EEG), electrooculography (EOG) and electromyography (EMG). Together with sensors measuring cardiac and respiratory activity, this sensor montage is collectively referred to as polysomnography (PSG).

Although it remains the gold standard for clinical assessment of sleep and diagnosis of sleep disorders, PSG is practically limited to one or two measuring nights, and cannot be effectively performed at home for a prolonged period of time. Over the last decade a variety of surrogate modalities have been studied to alleviate the cost and discomfort associated with polysomnography. One of the feasible surrogates is HRV acquired through cardiac sensors such as electrocardiography (ECG)^2–4^. HRV is a measure of autonomic nervous system activity^5^. The parasympathetic component of the autonomic system increases with sleep depth (i.e. N1, N2, N3) while the sympathetic component is related to awakenings. REM sleep is characterised by variations in the sympathetic to parasympathetic tone balance.

The inference of sleep stages is done by training machine learning algorithms which translate HRV features to sleep stages. The field has been increasingly studied in recent years. Most of the studies focused on sleep-wake classification^6–9^ and wake-REM-NREM classification^4,10–12^ while only a few have developed methods that separate light non-REM sleep (N1 and N2) from slow wave sleep (N3), i.e. wake-REM-N1/N2-N3 classification. The N3 class represents the most restorative period of sleep for metabolic functioning^13^ and is associated with maintenance of sleep and sleep quality^14^. Lack of N3 may have considerable impact on well-being, e.g., loss of daytime performance^14^. This work focuses on the 4-class classification problem of W-REM-N1/N2-N3 and the remainder of this section only reviews previous studies that have done that as well. Table 1 lists the best-performing methods published in recent past years.

**Table 1:** A list of best-performing methods for wake-REM-N1/N2-N3 classification (30-s basis) using autonomic activity.

Author, year	Participants	Sensors/signals	Algorithm	Cohen’s *κ*	Accuracy
Hwang^27^	12 healthy, 13 apnea	Bed sensors	Decision rules	0.48	70.9%
Tataraidze^33^	685 healthy	RIP	XGB	0.56	—
Beattie^22^	60 healthy	ACT, PPG	Linear discriminant	0.52	69.0%
Fonseca^30^	100 healthy	ECG, RIP	CRF	0.53	70.8%
Aggarwal^31^	400 apnea	Nasal flow	Neural CRF	0.57	74.1%
Li^25^	5793	ECG	Deep CNN	0.47	65.9%
This study	195 healthy, 97 patients	ECG	LSTM	0.61	77.0%

ACT: actigraphy, RIP: respiratory inductance plethysmography, ECG: electrocardiography, RF: radio frequency, XGB: extreme gradient boosting, CRF: conditional random field. CNN: convolutional neural network.

### Non-temporal models

Many algorithms have been published in the past that do not take into account temporal context when classifying sleep stages: in these models a set of *f* physiological features that are extracted for an epoch at time *t* in the night make up the feature space ${\mathscr{X}}={{\mathbb{R}}}^{f}$, with a marginal probability distribution *P*(*X*_*t*_). Together they form the domain ${\mathscr{D}}=\{{\mathscr{X}},P({X}_{t})\}$ of the sleep staging problem (note that no other epochs than the one at time *t* are included in the domain). The sleep stage label space ${\mathscr{Y}}$ then, in the simplified case of four-class sleep staging, comprises the labels $W,N1/N2,N3,R\in {\mathscr{Y}}$ (corresponding to Wake, combined N1 and N2, N3 and REM sleep) and the conditional distribution *P*(*Y*_*t*_|*X*_*t*_). The goal of the machine learning algorithm is then to find a solution for the classification task ${\mathscr{T}}=\{{\mathscr{Y}},P({Y}_{t}|{X}_{t})\}$. Performance is most often reported in accuracy and Cohen’s *κ*, a measure of agreement that factors out agreement by chance due to the imbalance in prevalence of different sleep stages throughout the night.

Some of the earlier ECG-based methods for sleep stage classification were published by Yilmaz *et al*.^15^ and Noviyanto *et al*.^16^. Noviyanto *et al*. found a random forest classifier to work best with Cohen’s *κ* of 0.43 and accuracy of 65.56% in a dataset of 18 participants. Yilmaz *et al*. found a support vector machine to perform best with with an accuracy of 73.1% (no 4-class Cohen’s *κ* reported) with 17 participants of which 5 with sleep apnea. More recently, Surantha *et al*.^17^ evaluated an approach using HRV features from ECG selected with a particle swarm optimization feature selection and a support vector machine (SVM) classifier, observing a similar accuracy of around 67% (Cohen’s *κ* was not reported).

HRV characteristics can also be derived from other sensors than ECG. Several studies validated photoplethysmography (PPG)-based approaches in identifying wake, sleep or REM sleep with acceptable performance^18–20^. To classify the four sleep stages, Hedner *et al*.^21^ used actigraphy, pulse oximetry, and peripheral arterial tone data from 227 apnea patients, and achieved a moderate performance with a Cohen’s *κ* of 0.48. In a recent study by Beattie *et al*.^22^, a large number (180) of motion-, breathing-, and HRV-based features were extracted from PPG and accelerometer signals obtained from 60 healthy adults. A linear discriminant analysis model was used in that study, achieving a slightly improved sleep staging performance (accuracy = 69%, Cohen’s *κ* = 0.52). de Zambotti *et al*.^23^ conducted a study including 44 adults to evaluate a commercially available device (Fitbit Charge 2), where REM sleep and Light sleep can be detected more reliably than wake and deep sleep. Fujimoto *et al*.^24^ attempted to classify sleep stages using a PPG sensor combined with a 3D accelerometer and they showed a classification accuracy of 68.5% based on data from 100 healthy volunteers. Most recently, Li *et al*.^25^ applied a deep convolutional neural network to ECG-derived spectrograms (as an alternative to hand-engineered feature extraction), and achieved a Cohen’s *κ* of 0.54/accuracy of 75.4% in a small validation hold-out (N = 18) and Cohen’s *κ* of 0.47/accuracy of 65.9% in a large dataset containing 5793 participants for 4-class sleep stage classification.

There were also studies that used autonomic characteristics of sleep other than HRV. Some notable works were presented by Hong *et al*.^26^ reported an accuracy of 81% using a Doppler radar system to capture cardiorespiratory activity; and Hwang *et al*.^27^ reported a Cohen’s *κ* of 0.48 and an accuracy of 70.9% using body movement and respiratory dynamics.

### Temporal models

Given that sleep architecture has common temporal patterns throughout the night, the non-temporal approach may not achieve optimal performance as it does not exploit the dependency between time steps. Short-term recurrent models solve this problem by formulating the classification task as ${\mathscr{T}}=\{{\mathscr{Y}},P({Y}_{t}|{X}_{t},{X}_{t-1})\}$. Adding the HRV characteristics of the previous time step *t* − 1 enables the model to learn the short-term epoch-to-epoch architecture of sleep. For example, they can capture the sleep stage dependent time-delay between cortical and autonomic nervous activities during transitions between some sleep stages (e.g. between light and deep sleep)^28,29^. A few methods have been proposed in this field. Fonseca *et al*.^30^ compared probabilistic classifiers using similar cardiorespiratory features and showed that a conditional random field classifier outperformed classifiers based on linear discriminant and hidden Markov models, with a Cohen’s *κ* and accuracy of 0.53 and 70.8% respectively for 100 healthy participants and of 0.45 and 69.7% respectively for 51 sleep apnea patients^30^. A structured learning approach with a neural conditional random field algorithm was recently proposed to identify sleep stages from nasal flow signals, where a Cohen’s *κ* of 0.57 was achieved^31^.

Given these improvements in performance, these approaches motivate the investigation of better temporal models that can take into account a wider temporal context, especially given the variance in sleep architecture as the night progresses^32^, making the relationship between *X*_*t*−1_ and *Y*_*t*_ variable throughout the night. A few approaches have been proposed in the past for this. Tataraidze *et al*.^33^ proposed to tackle time-varying patterns in sleep architecture through a cycle-based approach that adapts a priori probabilities over time for different sleep stages. Using an extreme gradient boosting algorithm on respiratory effort signals acquired from 685 participants, they improved the classification performance by 8% with a Cohen’s *κ* of 0.56 compared with their base algorithm. As an alternative, Fonseca *et al*.^4^ proposed learning the probability of each sleep stage for each epoch number of the night and using those probabilities to post-process the classification of the corresponding epochs. This approach was applied to the predictions of a linear discriminant classification approach with 142 HRV (measured from ECG) and respiratory effort features. The approach found a moderate overnight sleep staging performance (Cohen’s *κ* = 0.49, accuracy = 69%). While these solutions have shown empirical gain over non-temporal models, they are limited by the fact that they make an explicit connection between the time of the night and the expected sleep stages. It is easy to conceive of limitations of such methods. For example, disruptions during sleep may change the sleep architecture entirely, or insomnia patients might have an unusually long sleep onset period which these probabilities will fail to model as they are based on population statistics.

To overcome the issues of modelling sleep stage probabilities as a function of absolute time in bed, Willemen *et al*.^3^ proposed using contextual features based on an accelerometer, such as “time passed since the last observed movement” or “time until the next observed movement”. These relative measures combined with ECG and respiratory effort in an SVM method were used to classify the four sleep stages and a Cohen’s *κ* of 0.56 was achieved (however the epoch size with 60 seconds, unlike the 30 second epoch size used in other studies). This method is effective, however it is likely only capturing a fraction of the contextual information that could potentially help making better predictions. A more structural approach to temporal modelling is required that can model the task ${\mathscr{T}}=\{{\mathscr{Y}},P({Y}_{t}|{X}_{1},\mathrm{...},{X}_{t})\}$ for any given *t*, without being restricted to only short-term patterns, without relying on a priori assumptions based on the time in bed, and finally without being restricted to only a few features/characteristics of the data.

### Long short-term memory model

Bi-directional multi-level LSTM networks^34^ are temporal models that could potentially overcome all the limitations outlined in the last subsection, because they (1) can model temporal context unlike feed-forward approaches^17^, (2) have a large temporal scope unlike Markovian models such as conditional random fields^31^, (3) do not model class probabilities as a function of absolute time in bed^4^ and (4) can perform temporal inference over any feature, instead of being restricted to a set of hand-designed temporal features^3^, making them a promising solution for sleep stage classification. LSTM cells consist of memory units that can store long-term information from time series and generate an output based on the current time step input, their last output (short-term recurrence) and the internal memory state (long-term recurrence). The memory state is controlled through gating mechanisms. A detailed description and equations of LSTM cells are given in the original paper^34^. Stacking multiple layers of LSTM cells allows for the memorization of deeper temporal structures in the data. By having two LSTM stacks in parallel, one applied in the forward and another in the backward direction, it is possible to take into account both past and future input data to classify each single time step^35^, allowing the sleep scoring label to be conditioned on both the past and future epochs of the night. Such models could learn to capture both the desirable properties of short-term recurrent models, as well as model the temporal context of the night through memory cells, allowing it to reason over different contextual patterns independent of time slept.

LSTM-based algorithms have been applied in EEG-based sleep staging^36,37^ with excellent results, raising the question how long human annotation of sleep EEG will still be needed in the future. With non-EEG data, Sano *et al*.^38^ combined actigraphy, skin conductance, and skin temperature data using a LSTM method to enhance the performance of classifying sleep and wake. Zhao *et al*.^39^ proposed an adversarial architecture to LSTM to learn sleep stages (wake, REM, N1/N2, and N3) from radio signals from 25 healthy participants (annotated by an automated EEG sleep stage classifier) and achieved an unprecedented result (Cohen’s *κ* = 0.7).

LSTM approaches have not been applied to HRV-based sleep stage classification before. As mentioned earlier, Li *et al*.^25^ did use a deep neural architecture, but their model consisted of convolutional layers which serve to replace regular manually engineered features, rather than representing the long-term temporal dependencies in sleep stage scoring. Some tricks exist to increase the perceptive fields of convolutional networks (e.g. max pooling^25^), but it is always bounded to a limited time frame (comparable to how HRV features are extracted over windows of about 5 minutes, see Section 2.2). To the contrary, LSTM models can retain information in the internal cell state for a large number of discrete steps (> 1000), making them exceptionally good at long-term temporal modelling.

## Materials and Methods

### Materials

The data set used in this study was collected as part of the EU SIESTA project^40^ in the period from 1997 to 2000 in seven labs from five European countries. The study was approved by the local ethical committee of each research group. The ethical committees of the following departments have approved the study: Department of Psychiatry, School of Medicine, University of Vienna, Austria; Department of Neurology, School of Medicine, University of Vienna, Austria; Area d’Investigacio Farmacologica, Institut de Recerca de l’Hospital de la Santa Creu i Sant Pau, Barcelona, Spain; Department of Psychiatry, Free University of Berlin, Germany; Zentrum fur Innere Medizin, Klinikum der Philipps-Universitat Marburg, Germany; Department of Psychiatry, University of Mainz, Germany Department of Clinical Neurophysiology, Tampere University Hospital, Finland; Sleep Center, Westeinde Hospital, Den Haag, The Netherlands. All participants signed informed consent.The study was carried out in accordance with the relevant guidelines and regulations. Each participant was monitored for a total of 15 days and at day 7 and 8 participants were invited to sleep in the sleep laboratory to collect overnight PSG. The PSG included EEG, ECG, EOG and EMG measurements. Each recording was scored by two trained somnologists from different sleep centers according to the RnK guidelines^41^, and revised by a third expert who took the final decision in case of disagreement.

The total number of participants was 292, from whom 584 nights of recordings were collected comprising 541.214 annotated sleep epochs. Of those participants, 126 were female (252 nights, 43.3% of the data set). Participants had no history of alcohol use, drug use or irregular shift work. The data set further includes a total of 26 patients (52 nights) with insomnia disorder, International Classification of Diseases, tenth edition (ICD-10) F51.0. Insomnia was either related to a mild to moderate generalized anxiety disorder (ICD-10 F51.0) or mood disorder (ICD-10 F51.0 and F3). Furthermore, 51 patients (102 nights) were diagnosed with sleep apnea (ICD-10 G47.3), 5 patients (10 nights) with periodic limb movement disorder (ICD-10 G25.8), and 15 patients (30 nights) with Parkinson’s disease (ICD-10 G20)^40^. The total number of patients with a sleep or sleep-disturbing disorder was 97.

More details regarding participants and study design were described by Klosh *et al*.^40^. Table 2 contains participant demographics and sleep statistics.

**Table 2:** Demographics and sleep statistics of participants in the Siesta data set. Sleep statistics are computed based on the sleep stage annotation of the data set.

Parameter	Mean (SD)	Range
Age (year)	51.5 (17.3)	20.0–95.0
BMI (kg/m^2^)	25.6 (4.5)	16.5–43.3
TIB (hour)	8.0 (0.5)	5.8–9.6
SE (%)	80.8 (12.8)	14.6–99.1
N1 (%)	13.1 (8.4)	2.4–77.1
N2 (%)	53.8 (8.8)	13.6–78.8
N3 (%)	13.8 (8.4)	0.0–44.5
REM (%)	18.2 (5.9)	0.0–34.8

N1, N2, N3, and REM percentages were calculated over the total sleep time for each recording.

BMI: body mass index, TIB: time in bed, SE: sleep efficiency.

### Feature extraction

This study used a set of 132 HRV features extracted from inter-beat intervals (IBIs) computed from ECG. For this, a beat detection algorithm was used first to pre-process the signal to a sequence of IBI values. The algorithm was a modification^42^ of the Hamilton-Tompkins beat detection algorithm^43^. All features are summarized in Table 3 with citations to the original manuscripts.

**Table 3:** Cardiac features used in the study.

Count	Feature name
**50**	**Time domain features**
4	Means and medians of HR and RR (both detrended and absolute)^2,57^
12	SDNN, RR range, pNN50, RMSSD, and SDSD^57^, MAD^15^ (both detrended and absolute RR)
28	Percentiles (5%, 10%, 25%, 50%, 75%, 90% and 95%) of detrended and absolute HR/RR^15^
6	RR DFA, its short, long exponents and all scales, and WDFA over 330 s and PDFA over non-overlapping segments of 64 heartbeats^58–60^
**12**	**Frequency domain features**
4	RR logarithmic VLF, LF, and HF power and LF-to-HF ratio on 270 s windows^57,61^
4	Boundary-adapted RR logarithmic VLF, LF, and HF power and LF-to-HF ratio on 270 s windows^57,61^
4	RR mean respiratory frequency and power, max phase and module in HF pole^62^
**31**	**Entropy and regularity features**
20	Multiscale sample entropy 1 of RR intervals at length 1 and 2, scales 1–10 over 510 s^63^
1	Sample entropy of symbolic binary changes in RR intervals^64^
2	Short- and long-range phase coordination of R-R intervals in patterns of up to 8 consecutive heartbeats^7,11^
7	Phase synchronization for 6:2, 7:2, 8:2 and 9:2 phases, dominant ratio, short- and long-term coordination^46,47^
1	Higuchi’s fractal dimension of the normalized IBI sequence^48^
**39**	**Miscellaneous features**
21	Mean teager energy, % of transition points and maxima and mean and sd of intervals between them, mean and sd of the amplitude of normalized IBIs at transition points and maxima^44^
5	Arousal probabilities (max, mean, median, min, sd)^65^
13	Visibility graph features^49^

HR heart rate; RR R-R interval; SDNN standard deviation of RR; pNN50 pecentage of successive RR differences >50 ms; RMSSD, root mean square of successive RR differences; SDSD, standard deviation of successive RR differences; MAD, mean absolute difference; VLF, very low frequency; LF, low frequency; HF, high frequency; DFA, detrended fluctuation analysis; PDFA, progressive DFA; WDFA, windowed DFA; PSD, power spectral density.

^1^The estimation accuracy of sample entropy is lower in series shorter than 10^*m*^ (where *m* is the pattern length, in samples)^66,67^. In practice this means that this feature will be accurate for all scales with *m* = 1 and for scales below 6 with *m* = 2. The choice of window size was discussed in our earlier work^4^.

A large part of the feature set from these IBI sequences has been described in earlier work where a set of cardiac and respiratory features were evaluated^7^, however only the cardiac subset of the features is used in this work as no respiratory signal was included. The features were computed for each 30 second epoch of sleep by using a 4.5 minute window of heart beat data centred around the epoch (except when stated otherwise in Table 3). These were measures of HRV in the time domain, the frequency domain, results of entropy analysis, detrended fluctuation analysis, several measures of signal energy as well as features approximating the cardiorespiratory coupling during sleep by inspecting the regularity of the heart beat rhythm. Furthermore, Teager energy was used to characterize transition points and local maxima in IBI series^44^, including the mean energy, percentage of transition points and maxima, mean and standard deviation of intervals between transition points and maxima, mean and standard deviation of the amplitude of normalized IBI at transition points and maxima, all calculated based on the IBI time series, and on the first intrinsic mode function after empirical mode decomposition^45^.

To express the interaction between cardiac and respiratory autonomic activity, a cardiac-to-respiratory phase synchronization rate was determined by matching regular patterns in the sign of the IBI sequence. Patterns of 6:2, 7:2, 8:2, 9:2 are detected. The dominant rate is determined, as well as short- and long-term coordination in terms of presence and duration of synchronized heart beats^46,47^. Higuchi’s fractal dimension was also used as a measure of phase coordination^48^.

Finally, visibility graphs were used to model cardiorespiratory interaction in the IBI series^49^ and used to calculate the assortativity mixing coefficient, the mean and standard deviation of the clustering coefficients and degrees, slope of the power-law fit to the degree distribution and percentage of nodes with a small and with a high degree, all computed based on the visibility graph and the corresponding difference visibility graph^49^.

### Machine learning model

#### Model description

**Figure 1:**
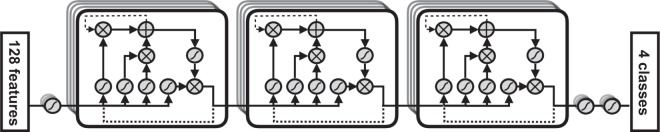
Computational graph of the neural network architecture. The three large blocks denote LSTM cells^34^. Dotted black lines denote recurrent connections that pass computed values to the next epoch in the sequence. The sigmoid-like functions are a linear combination of all inputs.

The model is illustrated in Fig. 1, showing how for a single epoch the features are translated into class probabilities. It consists of 5 layers: first, a perceptron layer; then three consecutive layers of LSTM cells; and finally 2 more perceptron layers. The first perceptron layer consists of 32 perceptrons, each generating a linear combination of all features. Each LSTM layer consists of 64 cells: 32 move in the forward direction, passing their internal values to future epochs, while the other 32 cells pass values in the opposite direction. Finally, the 64 values coming out of the last LSTM layer are processed by a perceptron layer with 32 neurones and subsequently a last layer with 4 neurones corresponding to the 4 class probabilities. All activation functions used are sigmoid, with the exception of the last layer where a softmax activation function is used.

#### Training and evaluation

The model is trained and validated using the Siesta database. The inputs are the HRV features computed from ECG and the labels were derived from the R&K annotations: S1 and S2 were combined as the “N1/N2” class and S3/S4 were combined as the “N3” class. The validation is done in a 4-fold cross-validation scheme in which folds are created on participant level, thus ensuring that nights from the same participants are always either in the training or the testing portion. An early stopping criterion was used to stop training the model once the loss on the test fold did not improve for a 100 subsequent passes over the training data. The model’s performance is quantified using performance over all folds.

For each fold, the 2.6·10^5^ free parameters of the model were trained simultaneously with the RMSprop optimizer, a variant of AdaDelta^50^ introduced in a lecture series^51^. Dropout^52^ on the input (20%), on LSTM outputs (50%) and LSTM recurrent connections (50%) was applied during the training phase to reduce overfitting. Categorical cross-entropy was used as the loss function during model fitting. Categorical cross-entropy *H*(*Y*,$\hat{Y}$) for a night with *N* epochs can be computed as:

1 $$H(Y,\hat{Y})=-\,\frac{1}{N}\mathop{\sum }\limits_{i=1}^{N}\,\mathop{\sum }\limits_{c=1}^{4}\,P({Y}_{i}={C}_{c})\cdot log(\hat{P}({Y}_{i}={C}_{c}))$$

where *C*_1_ up to *C*_4_ are the 4 sleep stages W, R, N1/N2 and N3, *P*(*Y*_*i*_ = *C*_*c*_) is the fraction of sleep annotators who scored *Y*_*i*_ as *C*_*c*_ and the *P*(*Y*_*i*_ = *C*_*c*_) is the model’s predicted probability (i.e. softmax activation) for the same epoch and class pair. By using this representation of the ground truth (instead of simply assigning a value of 1 to the consensus annotated class) the neural network can also learn to model the uncertainty between annotators.

#### Analysis

Performance is evaluated using metrics of accuracy and Cohen’s *κ* coefficient of agreement on an epoch-per-epoch basis in comparison with ground-truth. All performances are calculated per night, and both average and standard deviation are reported.

Performance is also analysed with respect to the demographic factors of age, sex and body-mass index (BMI). The effect of these demographic factors on accuracy and Cohen’s *κ* is tested through Pearson’s correlation coefficient for age and BMI and through a Mann-Whitney U-test between the two sexes. This test is used to avoid invalid results due to violations of parametric assumptions (e.g. normality) about the distributions of the performance that other tests may have. Next to that, performance for each of the main sleep disorder groups in the data set will be presented. Mann-Whitney U-tests are used to compare the performance distribution of each disorder to the healthy group.

Finally, performance will also be reported per sleep stage using several metrics of classification performance: precision (fraction of predicted instances of the class that was correct), recall (fraction of true instances of the class that were predicted as such), Cohen’s *κ* and accuracy.

#### Architecture optimisation

To optimise the size of the LSTM network, different model parameters were evaluated: models with 1 up to 6 LSTM layers were trained and compared, as well as models with 32, 64 and 128 cells per LSTM layer. For the best-performing model (3 LSTM layers and 64 LSTM cells per layer) the model was also evaluated without the use of any dropout, which showed a drastic decrease in performance. This is how the model configuration described in Sections 2.3.1 and 2.3.2 was chosen.

## Results

### Model training and evaluation

The 588 nights of the Siesta database^40^ were divided into 4 folds to evaluate the LSTM model. The data was randomly split into folds at the level of participants (294 in total). An overview of the data set is given in Table 2. For every 30 second epoch, all features from Table 3 were extracted, resulting in a feature sequence for every night, used as input to the machine learning model shown in Fig. 1. The test performance was obtained per fold by training the model on the remaining folds in a 4-fold cross-validation scheme. Performance in Cohen’s *κ* and accuracy is shown in Table 4. Alternative architectures with reduced and increased complexity as well as with and without dropout were also evaluated in the same manner. Performance for these models over all participants is shown in Table 4.

**Table 4:** Performance of the model over all folds, as well as the overall performance for alternative architectures.

Model	Nr of participants	Cohen’s *κ* ± sd	Accuracy % ± sd
***Performance for cross-validation of final model with 3 LSTM layers, 64 cells per layer***			
Fold 1	73	0.60 ± 0.15	76.53 ± 8.47
Fold 2	73	0.60 ± 0.14	76.28 ± 8.92
Fold 3	73	0.64 ± 0.15	78.60 ± 10.15
Fold 4	73	0.61 ± 0.16	76.58 ± 10.15
**All participants**	**292**	**0.61** ± **0.15**	**77.00** ± **8.90**
***Performance for clinical subgroups of final model with 3 LSTM layers, 64 cells per layer***			
Healthy subgroup	195	0.63 ± 0.16	76.53 ± 10.14
Sleep apnea subgroup	51	0.60 ± 0.15	78.50 ± 7.90
Insomnia subgroup	26	0.65 ± 0.14	78.50 ± 7.07
Parkinson’s disease subgroup	15	0.43 ± 0.17	65.38 ± 10.04
PLMD	5	0.62 ± 0.15	78.33 ± 4.81
***Performance for alternative models in 4-fold cross-validation, all participants***			
LSTM layer, 64 cells	292	0.59 ± 0.14	75.92 ± 8.64
LSTM layers, 32 cells	292	0.59 ± 0.15	75.54 ± 9.26
LSTM layers, 128 cells	292	0.61 ± 0.15	77.00 ± 9.02
LSTM layers, 64 cells	292	0.61 ± 0.14	76.64 ± 8.76

Performance for each of the sleep stages was also measured (precision, recall, accuracy, Cohen’s *κ*). For wake, precision was 0.73 ± 0.20, recall was 0.71 ± 0.20, accuracy was 0.90 ± 0.07 and Cohen’s *κ* was 0.63 ± 0.19. For REM, precision was 0.71 ± 0.22, recall was 0.76 ± 0.24, accuracy was 0.92 ± 0.04 and Cohen’s *κ* was 0.68 ± 0.22. For combined N1/N2, precision was 0.80 ± 0.11, recall was 0.82 ± 0.08, accuracy was 0.79 ± 0.08 and Cohen’s *κ* was 0.56 ± 0.15. Finally, for N3, precision was 0.62 ± 0.33, recall was 0.61 ± 0.30, accuracy was 0.92 ± 0.04 and Cohen’s *κ* was 0.53 ± 0.27. The prevalence of each of these sleep stages in the data is given in Table 2).

### The effect of demographic factors on performance

**Figure 2:**
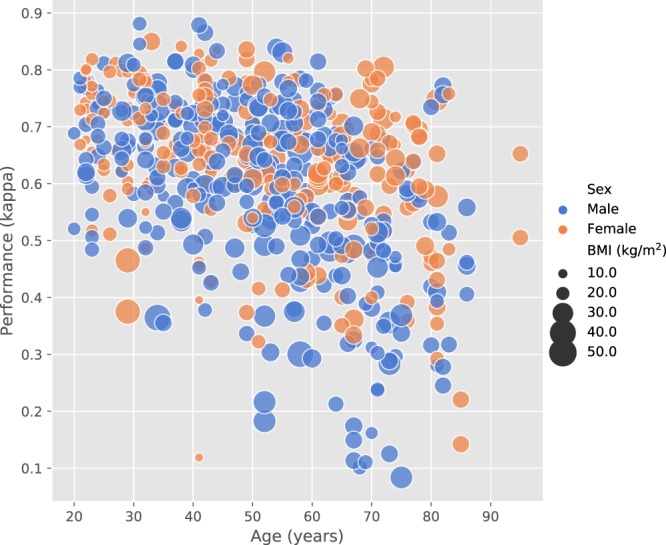
Distribution of Cohen’s *κ* over three key demographic factors: age, sex and BMI.

Subsequently, the test results of all folds were pooled together to analyze the performance over the entire data set. Relationships between demographic factors and Cohen’s *κ* performance are shown in Fig. 2. The correlation between demographic factors and performance was compared within the group of 195 healthy participants. Significant correlations were found for both Cohen’s *κ* (−0.45, *p* < 0.001, N = 584) and accuracy (−0.40, *p* < 0.001, N = 584) with age but BMI correlated with neither. There was a significant difference between the male (N = 126) and female (N = 252) subgroups for Cohen’s *κ* (*p* < 0.05, Mann-Whitney U-test) but not for accuracy.

**Figure 3:**
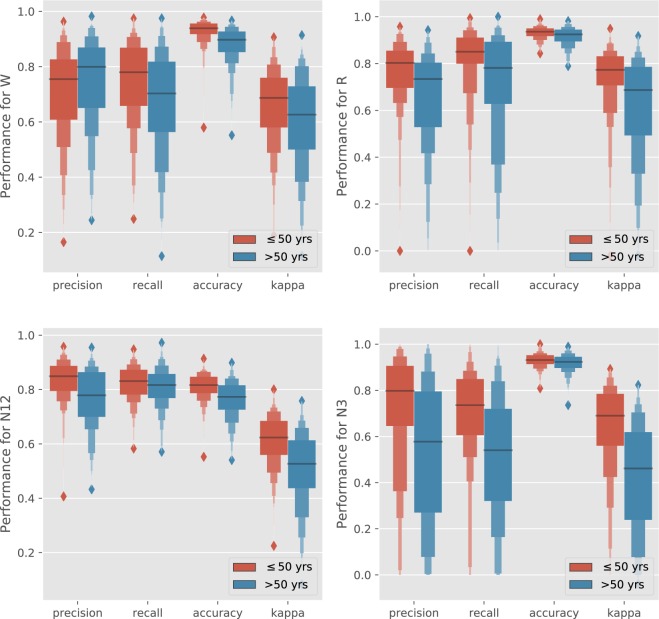
Distribution of performance for each sleep stage, reported in precision, recall, Cohen’s *κ* and accuracy, split across young and old age groups.

To understand which of the sleep stages are mostly affected by age difference, also the performance per sleep stage is shown across young (≤50) and old (>50) participants in Fig. 3. Performance is given using precision, recall, accuracy and Cohen’s *κ*. The differences between each of these metrics, for each sleep stage, across the two age groups, was compared in all 16 cases (Mann-Whitney U-test). All differences were significant (*p* < 0.05). The difference in recall for N1/N2 had *p* = 0.025, the differences for W precision, W Cohen’s *κ* and N3 accuracy had *p* < 0.01, the difference in accuracy for R had *p* < 0.001 while all other tests had a *p* value lower than 0.00001.

### Performance for different patient groups

**Figure 4:**
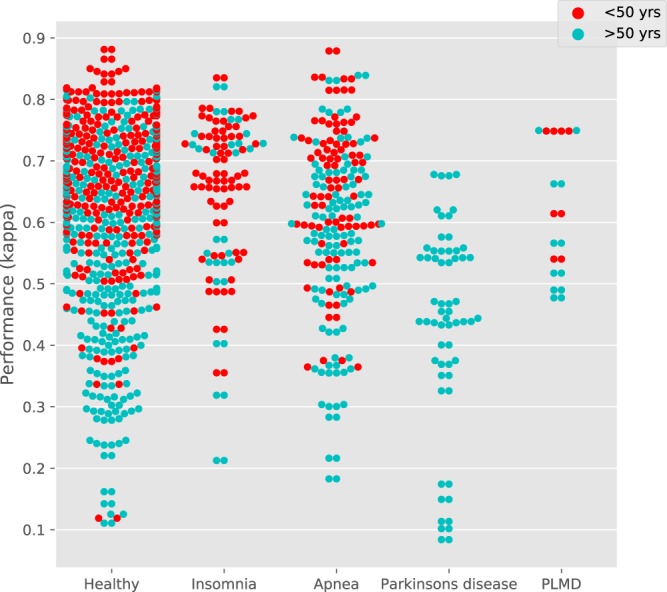
Distribution of performance in Cohen’s *κ* per patient group, broken down into young and old age segments. Every point represent a single night (multiple nights could belong to the same person).

The performance has also been assessed for each of the 5 most prevalent health profiles in the Siesta database, being patients of Sleep Apnea, Insomnia, Parkinsons disease, PLMD as well as a large Healthy control group. The distribution of the performances in Cohen’s *κ* for each of these groups is shown in Fig. 4 (broken down across ages) as well as summarized in Table 4.

Differences between the healthy group (N = 195) and the sleep disorder groups were tested for significance using a Mann-Whitney U-test on a participant level. Performance of Insomnia (N = 26) and Parkinsons disease (N = 15) patients did not differ significantly from Healthy. For Sleep apnea (N = 51), only Cohen’s *κ* had a significantly different performance (*p* < 0.05) while for PLMD (N = 5) both *κ* (*p* < 0.001) and accuracy (*p* < 0.001) were significantly different.

## Discussion

The presented model achieves state-of-the-art performance for HRV-based sleep stage classification, surpassing all previously published results presented in the introduction and summarized in Table 1, even though a significant part of the dataset used to evaluate this model included participants with disorders and old age, which are rarely included in prior work. These results confirm the capabilities of deep sequence-to-sequence models such as LSTM for sleep stage classification, in comparison to traditional feed-forward or short-term recurrent approaches. The obtained performance was also higher than what was obtained by Li *et al*.^25^ (Table 1) who evaluated a convolutional neural network model using ECG data in a very large dataset. As explained in Section 1.3, convolutional neural networks, while also falling in the same category of “deep neural networks”, do not address the long-tem temporal dependencies that LSTM models address, instead their main function is to replace manual, local, feature extraction. Thus, while it may be an attractive alternative to manual feature engineering, it does not fulfil the same function as the LSTM model. Future work should aim at combining a deep convolutional (or similar) structure to replace feature engineering with a LSTM (or similar) structure to capture the long-term temporal dependencies, however this would require an extremely large amount of data that has not been available for this purpose so far.

A non-EEG approach where LSTMs have been used is the work of Zhao *et al*.^39^ where they achieved a Cohen’s *κ* of 0.70, higher than what we have seen. However the input modality used in that study (radio-frequency signals) potentially contains more information (such as posture). The model was also only trained and evaluated in 25 participants, leaving generalisability as an unknown.

The performance of the model was examined across demographic factors as well as patient profiles. Figure 2 illustrates the negative correlation found between performance and age. This finding resonates further in the examination of patient profiles: while performance for insomnia, sleep apnea and PLMD patients remained close to the healthy control, a strong (and significant) drop in performance was observed for Parkinson’s disease patients. In Fig. 4 it is seen that all Parkinson’s disease patients were over 50 years old, thus partially explaining the short-coming of the method for this patient profile. In the same figure, it is clearly visible that in other groups, including the healthy group, performance tends to be lower for those over 50 years of age. However a different explanation for the decrease in performance for Parkinson’s disease patients could be the autonomic dysfunction associated with the disease^53^.

In Fig. 3 an overview of performance was given for each individual sleep stage, again split up at the age of 50. Performance drops are most apparent for the N3 and R classes across these age segments, while performance distributions of were closer to each other for the two age segments. However all differences between the two age groups were significant. The performance decrease for participants of higher age are likely caused by the changes in both autonomic function^54,55^ as well as sleep architecture^56^ with older age. Further research is needed into this age group, especially focused on improving performance for the non-wake classes. Auxiliary training objectives could be used to steer the neural network towards a better performance in this age demographic, for example by amplifying the neural network’s loss function for older participants or by steering the model to learn an age-invariant representation of the data.

## Conclusion

In conclusion, the method presented in this study performs at a level that advances the state-of-the-art for HRV-based sleep stage classification. However there are still issues to be addressed until the system can reach the accuracy of EEG-based sleep stage classification systems. Especially performance in older individuals remains limited. Complementing the HRV data with other unobtrusive modalities or improving the neural architecture through auxiliary learning tasks and other regularizers could both enhance performance. In the meantime, the method could still have a variety of clinical uses as a low-burden, cheap alternative to home polygraphy in low acuity settings. Finally, the pathological profiles in the data set do not fully represent the clinical population and thus further validation in larger clinical populations is also warranted.
